# A Comparative Analysis of Cold Brew Coffee Aroma Using the Gas Chromatography–Olfactometry–Mass Spectrometry Technique: Headspace–Solid-Phase Extraction and Headspace Solid-Phase Microextraction Methods for the Extraction of Sensory-Active Compounds

**DOI:** 10.3390/molecules29163791

**Published:** 2024-08-10

**Authors:** Esteban Narváez, Esteban Zapata, Juan David Dereix, Carlos Lopez, Sandra Torijano-Gutiérrez, Julián Zapata

**Affiliations:** 1 Laboratory of Residue Analysis, Faculty of Exact and Natural Sciences, University of Antioquia, Calle 67 No. 53-108, Medellín 050010, Colombia; esteban.narvaez@udea.edu.co (E.N.); juan.zapata77@udea.edu.co (E.Z.); juan.dereix@udea.edu.co (J.D.D.);; 2 SINBIOTEC Research Group, School of Engineering and Basic Sciences, University EIA, Calle 25 Sur # 42-73, Envigado 055428, Colombia; sandra.torijano@eia.edu.co

**Keywords:** cold brew (CB), aroma, flavor, coffee, headspace–solid-phase microextraction (HS-SPME), headspace–solid-phase extraction (HS-SPE)

## Abstract

Coffee, one of the most widely consumed commodities globally, embodies a sensory experience deeply rooted in social, cultural, and hedonic contexts. The cold brew (CB) method, characterized by cold extraction, is a refreshing and unique alternative to traditional coffee. Despite its growing popularity, CB lacks defined preparation parameters and comprehensive analysis of its aromatic composition. In this study, we aimed to obtain a representative extract of the volatile matrix of CB and characterize the aroma of sensory-active compounds using advanced techniques such as headspace–solid-phase Microextraction (HS-SPME) and headspace-solid-phase extraction (HS-SPE) for volatile compound extraction, followed by gas chromatography–olfactometry–mass Spectrometry (GC-O-MS) for compound identification. Optimization of the HS-SPME parameters resulted in the identification of 36 compounds, whereas HS-SPE identified 28 compounds, which included both complementary and similar compounds. In HS-SPME, 15 compounds exhibited sensory activity with descriptors such as floral, caramel, sweet, and almond, whereas seven exhibited sensory activity with descriptors such as chocolate, floral, coffee, and caramel. This comprehensive approach to HS-SPME and HS-SPE aroma extraction with GC-O-MS offers an efficient methodology for characterizing the aroma profile of CB, paving the way for future research and quality standards for this innovative coffee beverage.

## 1. Introduction

Coffee is a widely consumed product worldwide and is the second most consumed commodity in international markets [1]. These attributes are due to various qualities, such as caffeine content, its role in social life, aroma, and flavor. These combined factors confer a hedonic character of great scientific interest in coffee, comparable to wine and beer, which has motivated the search for new ways to consume coffee and generate different experiences [2].

The aroma and taste of coffee are fundamental parameters for evaluating its quality. Preparation methodology plays a crucial role in extracting compounds with sensory activity. Among these, cold brew (CB) stands out as a cold extraction technique that has experienced significant growth in recent years owing to consumers’ quest for new sensory experiences [3]. CB is a coffee preparation technique known for its fresh and smooth flavor compared to traditional heat methods. Two methodologies have become famous for CB preparation: the cold drip system, which allows continuous water renewal, and cold immersion systems, in which coffee is directly immersed in water [4].

Regarding sensory attributes, CB is often described using flavor terms such as chocolate, sweet, cocoa, fruity, and caramel. This technique reduces the acidity and bitterness of traditional hot coffee preparation methods [1,5]. Regarding caffeine, a compound of high interest in coffee owing to its various properties [6], it has been found that in CB, owing to prolonged extraction times, the caffeine content increases compared to other conventional preparations [7]. Chlorogenic acid content, directly related to antioxidant capacity, decreases in CB because of the lower temperatures in their preparation, as these compounds are less soluble in water [8].

Substances such as pyrazines, furans, and phenols have also been identified [9]. However, a comprehensive analysis of volatile compounds that are highly relevant to coffee aroma on a widespread basis has not yet been conducted. To date, comparisons have been made between extracted volatile compounds and information available in the literature [1]. Nevertheless, it is essential to note that olfactometric analyses cannot wholly replace instrumental analyses because various variables can affect a compound’s aroma [10,11,12].

Based on the above and considering that a clear definition has not been established or suggestions have been provided on optimal conditions and practices for CB preparation, our main objective was to develop a method for extracting and identifying volatile compounds with sensory activity present in CB. To achieve this, we propose a combination of techniques, such as gas chromatography coupled with olfactometry and mass spectrometry (GC-O-MS), adopting a comparative perspective and headspace-solid-phase extraction (HS-SPE) and headspace–solid-phase microextraction (HS-SPME) methods [6,12,13]. These sample preparation methods were chosen because of the complementary nature of the techniques used for the aroma analysis. This approach allowed us to obtain a more comprehensive characterization of the volatile compounds in the cold brew and their contribution to the sensory profile of the final product.

## 2. Results

### 2.1. Optimization of the HS-SPME Technique

**Figure 1:**
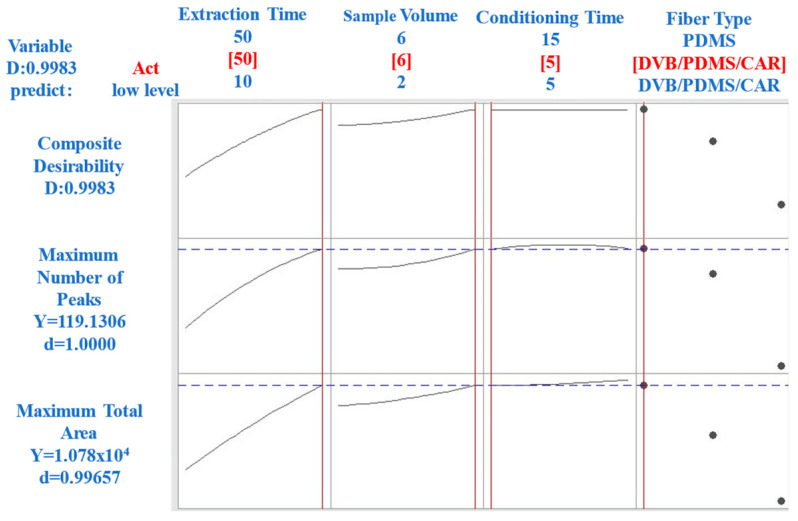
Optimization of HS-SPME variables (Ext.time: extraction time, Cond.time: conditioning time, Sample Vol.: sample volume, and Fiber: fiber type) based on response factors (total area and number of peaks).

Figure 1 shows the optimization process for the HS-SPME technique, analyzing the variables of extraction time, conditioning time, sample volume, and fiber type based on the response factors (total area and number of peaks). The goal was to identify the optimal conditions for extracting volatile compounds from CB coffee.

Different extraction times were evaluated to determine the time required to reach an optimal equilibrium between the volatile compounds and the HS-SPME fiber. As seen in the first plot of Figure 1, the response factor (total area) increased with time and plateaued at approximately 50 min. Therefore, an extraction time of 50 min was chosen to ensure maximum extraction efficiency. The second plot in Figure 1 illustrates the effect of conditioning time on the response factors. A conditioning time of 5 min was sufficient to stabilize the sample matrix without a significant loss of volatiles, as indicated by the steady response factor after this time. The third plot in Figure 1 depicts the relationship between the sample volume and response factor. A volume of 6 mL was determined to be optimal, providing enough sample for effective volatile extraction while maintaining practical handling and analysis conditions. The type of fiber used in HS-SPME can significantly affect the efficiency of volatile extraction. The fourth plot in Figure 1 compares the performance of the fibers with different polarities. The DVB/PDMS/CAR fiber was the most effective, owing to its mixed polarity characteristics, capturing a broad range of volatile compounds.

In summary, the optimal parameters for the HS-SPME technique in this study were a 50-min extraction time, a 5-min conditioning time, a sample volume of 6 mL, and Divinylbenzene/Carboxen/Polydimethylsiloxane (DVB/CAR/PDMS) fibers. These conditions provided a comprehensive and representative profile of volatile compounds in CB (Figure 1).

#### 2.1.1. Type of Fiber

**Figure 2:**
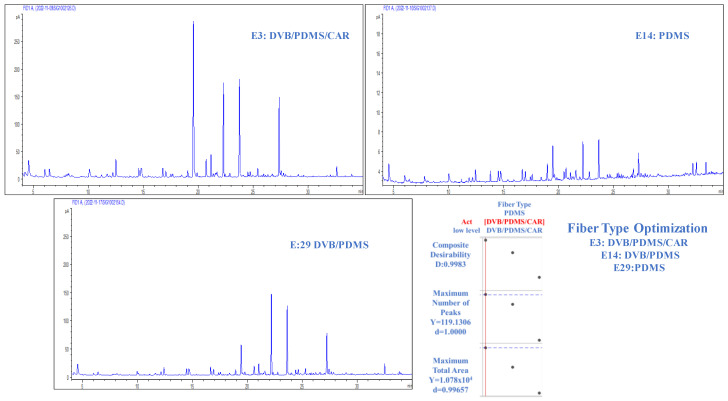
Chromatograms obtained from experiments E3 (extraction time = 50 min; sample volume = 4 mL; conditioning time = 15 min; fiber type = DVB/PDMS/CAR); E14 (extraction time = 50 min; sample volume = 4 mL; equilibrium time = 15 min; fiber type = PDMS); E29 (extraction time = 50 min; sample volume = 4 mL; equilibrium time = 15 min; fiber type = DVB/PDMS) with the fiber type optimization graph.

The extraction capacity of the volatile matrix of CB was evaluated using three types of fibers (DVB/PDMS/CAR, DVB/PDMS, and PDMS), as shown in Figure 2. One of the crucial aspects in optimizing the HS-SPME technique is the choice of extraction phase or fiber, as this stage plays a fundamental role in the essential equilibria that guide the extraction process. Within this framework, special attention has been paid to the choice of coating [3].

This study selected the DVB/PDMS/CAR extraction phase based on the chromatographic signal performance. Compared with the other evaluated phases, this combination exhibited more detected signals and a more prominent signal intensity. The corresponding chromatograms are shown in Figure 2. Experiments E3, E14, and E29 (Table S1) were analyzed, and all variables were kept constant except for the type of fiber. This result demonstrates that the fiber choice significantly influences the number of peaks and the total area of the extracted compounds.

In experiment E14, where the PDMS (non-polar) fiber was used, a lower extraction of analytes and their respective concentrations was observed, indicating a lower affinity for specific compounds. In contrast, for DVB/PDMS/CAR and DVB/PDMS fibers with different polarities, broader and more varied extractions of the compounds were achieved. Experiment E3, which used the triple fiber (DVB/PDMS/CAR), yielded the highest response parameters compared with the other fibers. This position is the most suitable option for the extraction methodology because it can extract compounds with various polarities, as its coating is composed of three types of phases with different characteristics [1,3,14,15,16,17,18]. This quality is consistent with findings in the literature describing the volatile composition of coffee preparations, indicating the presence of compounds with a wide range of polarities. Additionally, several extraction methods proposed in similar studies agree on the efficacy of triple fibers [1,19,20].

This detailed analysis of the type of fiber used provides a solid basis for selecting DVB/PDMS/CAR FIBER as the preferred option for extracting volatile compounds from CB.

#### 2.1.2. Extraction Time

**Figure 3:**
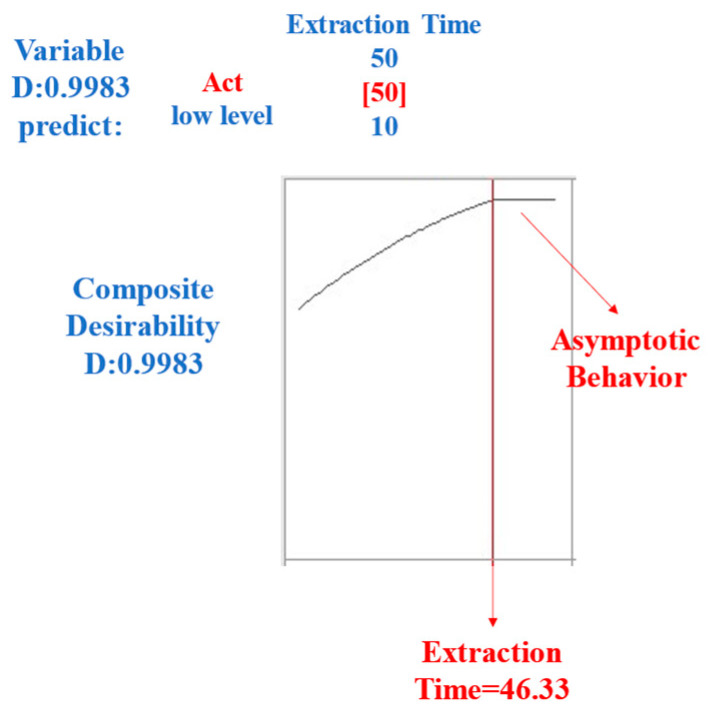
The optimization of the extraction time for HS-SPME was analyzed based on the response factors (total area and number of peaks).

The optimization of the extraction time is a central aspect of this study, and its detailed analysis provides an illuminating perspective on the optimal value to consider. Figure 3 shows the graph’s asymptotic behavior, starting at 46 min of extraction. This phenomenon suggests that the differences in extraction yields were insignificant when the extraction time exceeded 50 min.

**Figure 4:**
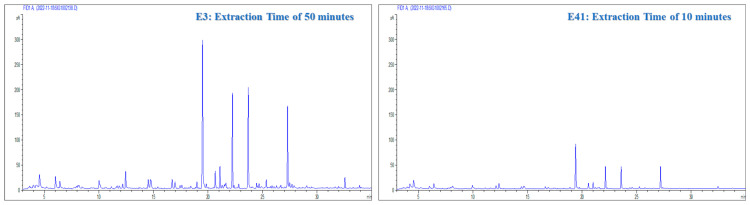
Chromatograms obtained from experiments E3 (extraction time = 50 min; sample volume = 4 mL; conditioning time = 15 min; fiber type = DVB/PDMS/CAR) and E41 (extraction time = 10 min; sample volume = 4 mL; conditioning time = 15 min; fiber type = DVB/PDMS/CAR) with interaction graphs of extraction times for the total area and number of peaks.

The influential statistical correlation associated with extraction time is based on the chemical equilibria during extraction. This central characteristic makes extraction time a critical variable for optimization. During this period, volatilization processes and the interaction of analytes with the coating of the extraction fiber were triggered. Figure 4 illustrates the relationship between extraction time and chemical processes. This figure keeps all of the variables constant except for the extraction time. The substantial changes observed in the interaction graphs during the different extraction times clearly show the significant influence of this variable by causing notable adjustments in the quantity and concentration of the extracted compounds.

Understanding the chemical equilibria involved in the extraction process highlights the need to consider this variable carefully. The complex relationship between absorption, volatilization, and interaction with the extraction fiber directly influences the quantity and concentration of the extracted compounds. This delicate process seeks to reach the point where extraction is optimally balanced, thus ensuring the effective retrieval of volatile compounds in the CB.

In summary, extraction time plays a critical role in optimizing the HS-SPME technique, and its influence on the results and intricate relationship with the underlying chemical processes demonstrate its fundamental role in obtaining representative and precise samples.

#### 2.1.3. Sample Volume Analysis

The analysis of the sample volume is a critical component of the optimization process. Although the statistical influence was not highlighted, the significance of the results is undeniable. The optimal sample volume was determined to be 6 mL. This choice arises as a strategic balance to achieve representative and accurate results.

An optimal value of 6 mL was established to maximize performance and avoid unwanted interactions between the sample and the fiber. Contact between the sample and the fiber could lead to the inclusion of non-volatile compounds in the fiber or a significant decrease in the headspace. It is essential to emphasize that such interactions could potentially alter the results, deviating from the accurate volatile composition of CB [21].

It is important to note that the choice of this value not only avoids immersion problems, which are characteristic of different HS-SPME methodologies. but addresses the inverse relationship between sample volume and headspace. As the sample volume increased, the headspace available for the volatilization of the compounds decreased. Consequently, the number of analyses that could be extracted from the headspace is reduced.

This inverse relationship between the sample volume and the headspace can be explained by Equation (1) [17]:

(1) $${{n=C}_{e}^{\infty }v}_{e}=C_{o}\frac{k_{sh}v_{s}v_{e}}{k_{sh}v_{e}+v_{s}}.$$

where n represents the number of moles, $C_{o}$ is the concentration of the analyte in the sample before extraction, $v_{e}$ is the volume of the equilibrium phase, is the concentration of the analyte once equilibrium is reached in the equilibrium phase $C_{e}^{\infty }$, $k_{hs}$ is the equilibrium constant a⇋b for the solid phase, $v_{s}$ is the volume of the solid phase, and $v_{m}$ is the sample volume.

The equation establishes that the number of moles (*n*) is directly related to the sample volume and the concentration of volatile compounds in the headspace. As the sample volume increased, the other factors in the equation tended to approach one, increasing the concentration of analytes in the headspace.

The optimal choice of 6 mL for the sample volume is presented as a coherent result with the literature. It supports the goal of obtaining representative and accurate results using HS-SPME methodology.

#### 2.1.4. Conditioning Time

Conditioning time, which focuses on increasing the sample temperature to 37 °C, emerged as an essential component of the optimization process. Although there is little variability in the recorded times, it is crucial to understand that this is because the samples did not require extremely high temperatures for proper conditioning.

A relevant observation is shown in Figure 1, representing the consistent equilibrium time behavior in HS-SPME methodologies with prolonged extraction times. This finding allows us to address conditioning time from the economic and operational efficiency perspective.

Figure 1 shows the stability of the equilibrium time, even when prolonged extraction times were applied. This finding opens the door to the possibility of selecting conditioning times as low as 5 min without compromising the efficiency of the process. This shorter conditioning time can improve the methodology’s economy without compromising performance.

In summary, the conditioning time with the central objective of reaching a temperature of 37 °C aligned harmoniously with the requirements and operational considerations of the sample. Even with prolonged extraction times, it is feasible to choose shorter conditioning times, contributing to the economic efficiency and effective functioning of the HS-SPME methodology.

### 2.2. Verification of Aroma Extraction Methodology through HS-SPE

The results of the HS-SPE technique confirm that a 4-h extraction is optimal for capturing the aroma of CB, achieving a balance between the quantity and quality of the extracted compounds. A purge gas flow rate of 100 mL/min was used to demonstrate the efficient extraction. Additionally, the elution solvent ratio of dichloromethane/methanol (95/5) was identified as optimal for eluting analytes, and these findings coincide with those of previously established methods [22].

#### 2.2.1. Extraction Time

The extraction time significantly impacted the chromatographic results, influencing the quantity and area of the volatile compounds in the CB sample. For example, when the extraction time was brief (e.g., one hour), incomplete extraction of all compounds of interest in the sample was possible, affecting both the concentration and quantity of the extracted compounds and the quality of the chromatographic results.

However, when the extraction time is excessively extended, as in the case of 6 h, degradation of compounds, desorption of compounds, or loss of volatile analytes may occur (Table 1).

The results suggest that 4 h extraction emerges as the most pertinent option, as it proves to be more stable concerning the provided data. This extraction duration balances the determination of a wide range of volatile compounds of interest while preserving their integrity and the quality of the chromatographic results.

#### 2.2.2. Purge Gas Flow

The effects of three different purge gas flow levels were evaluated: 80 mL/min, 100 mL/min, and 120 mL/min. A base flow rate of 100 mL/min was initially established, and from this value, the flow rate was varied between 80 and 120 mL/min.

The optimization of this variable significantly influenced the extraction of the compounds of interest. An excessively fast purge gas flow could reduce the contact time between the sample and the purge gas, decreasing the extracted compounds. In contrast, slow purge gas flow can increase the contact time with the sample, potentially increasing the amount of impurities extracted or weakening the retention of the compounds.

The results, detailed in Table 2, reveal that despite the levels evaluated in the purge gas flow, there was no significant impact on the areas and number of peaks in the chromatographic results. Although the purge gas flow rate did not significantly influence the chromatographic results, maintaining a flow rate of 100 mL/min was considered the most appropriate. This value balances the efficiency of extracting representative compounds from the matrix and minimizing impurities in the chromatographic results.

#### 2.2.3. Solvent Ratio in the Elution of Target Analytes from the Sorbent Material

The solvent ratio used in the elution of target analytes from a sorbent material is a critical factor in solid-phase extraction (HS-SPE). In this process, a stationary phase or sorbent material, in this case, a LICHROLUT EN resin packing, is used to retain the target analytes present in the sample selectively. The choice of stationary phase and solvent ratio in elution is based on the polarity and chemical properties of the analytes, as well as the characteristics of the sample matrix.

The elution solvent must be capable of efficiently desorbing analytes from the sorbent while minimizing interference from other components in the sample. Using an appropriate elution solvent significantly improves the recovery and selectivity of the extraction process.

The solvent ratio can affect the representativeness of extracts obtained from the matrix. Increasing the polarity of the elution solvent mixture does not always result in greater elution of polar analytes. It results in a stronger retention of non-polar analytes on the sorbent, leading to inefficient elution. Moreover, increasing the polarity of the elution solvent can intensify interference from other sample components that co-elute with the target analytes, thus compromising the selectivity of chromatographic separation. Therefore, selecting an elution solvent with optimal polarity for the analytes and the sorbent is crucial.

The results are detailed in Table 3, which shows that the optimal solvent ratio for the elution of analytes, which demonstrated the best results, was 95/5, aligned with the initially employed methodology.

### 2.3. Identification of Compounds with Sensory Activity Using GC-O-MS

**Figure 5:**
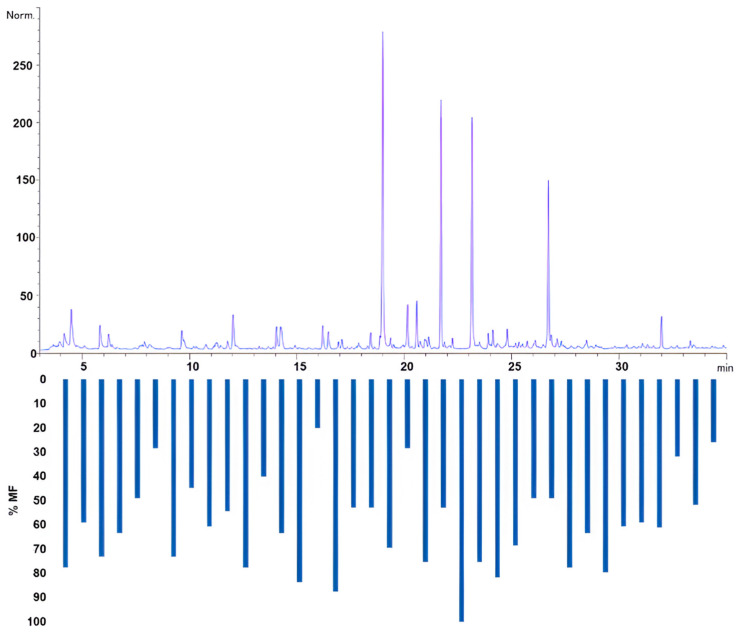
Olfactogram obtained through HS-SPME-GC-O-MS with Modified Frequencies.

Compounds with aroma-sensory activities were comprehensively identified using HS-SPME and HS-SPE methods with GC-O, where we obtained the olfactogram of the samples of CB (Figure 5). The combination of these techniques allowed for the tentative identification of these compounds, shedding light on their presence and possible impact on the sensory properties of CB.

The bar graph in Figure 5 illustrates the modified frequencies (%MF) of the compounds identified using HS-SPME-GC-O-MS. Each bar represents a compound with sensory activity, and the height of the bar indicates the modified frequency percentage. The higher the %MF, the more frequently the compound was detected in the olfactometric analysis, suggesting its prominence in the aroma profile of CB. Compounds with higher %MF values will likely significantly impact the overall sensory experience. The olfactogram provides a visual representation of the sensory-active compounds and their relative significance based on the frequency of detection and the intensity of the aromatic signal, thereby offering a means of assessing their importance in the aroma of CB.

Table 4 details the results obtained for compound identification. Each identified compound was presented with its IUPAC name and corresponding CAS registry number. Additionally, experimental Kovats retention index values (RI(E)) are provided, offering information on the elution and interaction of the compounds with the chromatographic column.

The experimental retention indices (RI(E)) were compared to those from the NIST19 Library (RI) to ensure the validity and consistency of the results. This comparison allowed for the evaluation of each compound’s precision and identification quality.

A total of 45 compounds with sensory activity were extracted using these techniques, of which 42 were tentatively identified (Table 4). Most of these compounds are detected in traditional coffee beverages [9,17].

The results revealed the presence of a variety of compounds, both common in traditional coffee beverages and less common or present at low concentrations. These compounds offer aromatic notes ranging from fruity to sweet and phenolic compounds. With approximately fourteen compounds with sweet notes, nine toasted, seven fruity, seven woody, five floral, and five green, the documented information in the literature is reinforced, highlighting the sensory affinity of CB with sweeter and fruitier aromatic notes, as well as the absence of burnt notes. The identification of these sensory-active compounds not only establishes a solid foundation for understanding the aromatic profile but also contributes to advancing knowledge in this research field.

Compounds with significantly high modified frequency percentages, such as ethanone, 1-(2-furanyl), ethanone, 1-(2-thienyl), γ-Butyrolactone, 2-Buten-1-one, 1-(2,6,6-trimethyl-1,3-cyclohexadien-1-yl), 1*H*-Pyrrole, 1-(2-furanyl methyl), were identified. These compounds are not typical in coffee and are found at deficient concentrations. It is hypothesized that this phenomenon could be related to the high quality of coffee, or the preparation methodology used, which could facilitate the extraction of these uncommon compounds. Other analytical methods are necessary to obtain more conclusive results regarding these phenomena, and an additional methodology should be established to quantify the presence and concentration of these compounds more precisely [2].

Another relevant observation is the detection of compounds with fruity, sweet, green, and other descriptors, such as linalool oxide/(*Z*)-linalool oxide, Furfural, Ethanone, 1-(2-furanyl), benzaldehyde, 5-methylfuran-2-carbaldehyde, Butyrolactone, 2-Buten-1-one, 1-(2,6,6-trimethyl-1,3-cyclohexadien-1-yl), Phenol, 2-methoxy, Benzyl alcohol, Furan, 2,2′-[oxybis(methylene)]bis, 1H-Pyrrole-2-carboxaldehyde, and 2-Methoxy-4-vinylphenol, among others. These compounds are common in fruit, wine, and liquor matrices. These findings align with literature assertions describing CB with fruity, sweet, chocolatey, and vinous notes [1,5].

Finally, the one retention time of the aromatic signals could not be assigned to the specific compounds. There were two main reasons for this finding. First, none of the results obtained from the NIST19 database showed retention indices close to those of the chromatograms. These compounds are relatively rare in the aromatic composition of coffee. This result could be attributed to the limited resolution of the mass detector and the relatively common mass/charge ratios (*m*/*z*) of these compounds, which make their precise assignment difficult. Additionally, two of these compounds had modified frequency percentages (%MF) below 50%, indicating that their relevance to the aroma of CB was limited. The compounds identified by comparing the mass spectra and the NIST19 database are shown in Table S4 [10].

A comparison of HS-SPME and HS-SPE extraction techniques revealed an intriguing complementarity in compound identification. Sixteen compounds were identified, which were only extracted and detected using HS-SPME, whereas seven exclusive compounds were identified using HS-SPE (Table 5). This finding underscores the diversity and specificity of each technique for extracting different components present in the aroma of CB.

It is essential to highlight that the choice between these techniques should not be interpreted as competition but as a complementary strategy. HS-SPME has been proven to be particularly effective in this context by identifying a more significant number of unique compounds. However, this does not diminish the relevance of the HS-SPE, which also provides unique information. Together, these techniques offer a more comprehensive and detailed understanding of the aromatic complexity of CB, synergistically contributing to our knowledge of the sensory-active compounds in this beverage.

It is worth noting that HS-SPME excels in efficacy and meets green chemistry parameters by avoiding solvents, demonstrating greater practicality, and requiring less analysis time than HS-SPE.

## 3. Materials and Methods

### 3.1. Raw Materials

The coffee used in this study was obtained from micro-batches and grown following a traditional milling process encompassing fermentation, washing with source water, and traditional drying in the sun. This coffee of the Castillo Arabica variety is grown at 1300 m above sea level. Additionally, the samples were subjected to medium roasting and grinding.

### 3.2. Sample Preparation

Preparation was carried out by employing immersion methodology, weighing 8.00 ± 0.20 g of coffee per 100 mL of water. The water used was purified Cristal brand water with a pH of approximately 7. The mixture was left to rest at a temperature between 2–4 °C for 15 h, and finally, it was filtered using a number 2 filter designed for coffee makers.

### 3.3. Reagents and Supplies

The alkane blend ranged from C6 to C30 (Supelco, Bellefonte, PA, USA). Analytical quality water was obtained using a MilliQ^®^ purification system (Merck KGaA, Darmstadt, Germany). Three types of fibers were used in the extraction process: polydimethylsiloxane/divinylbenzene/carboxeno (PDMS/DVB/CAR), polydimethylsiloxane (PDMS), and divinylbenzene/polydimethylsiloxane (DVB/PDMS), each with a length of 2 cm (Supelco, Bellefonte, PA, USA). Cartridges with an internal diameter of 0.8 cm and an internal volume of 3 mL filled with 400 mg of LICHROLUT EN (Merck KGaA, Darmstadt, Germany) resin were used for HS-SPE. The solvents used were dichloromethane (99.5% purity; LOBA Chemical, Mumbai, India), methanol (Merck KGaA, Darmstadt, Germany), and hexane (Merck KGaA, Darmstadt, Germany).

### 3.4. Chromatographic Conditions (GC-MS and GC-O-FID)

Chromatographic conditions were established based on previous studies on CB with modifications [14]. It was possible to determine the optimal chromatographic conditions for obtaining the chromatographic profiles of the volatile compounds in the CB. In the analysis, a Trace 1300 chromatography system coupled with a TWQ Duo Mass Spectrometer (Thermo Fisher Scientific, Waltham, MA, USA) was used. In addition, the NIST library was used to compare the mass spectra.

The injections were performed using a split/splitless injector in splitless mode at a temperature of 220 °C, pressure of 298 kPa, and total flow of 83 mL/min. The temperature program was as follows: an initial temperature of 50 °C was set for 3 min and then increased at a rate of 3 °C/min until it reached 150 °C. The temperature was then increased at 7 °C/min to 240 °C and maintained for 2 min. A 60 m long DB-FFAP column with an internal diameter of 0.25 mm and a film thickness of 0.25 μm was used (Agilent Technologies, Palo Alto, CA, USA). For the optimization of HS-SPME, verification of HS-SPE, and olfactometric analysis, a 6890N gas chromatograph (Agilent Technologies, Palo Alto, CA, USA) equipped with a flame ionization detector (FID) coupled to an Olfactory Detection Port (ODP2, Gerstel, Mülheim, Germany) was used. A DB-WAX column (30 m, 0.25 mm internal diameter, and 0.5 μm film thickness, Agilent Technologies, Palo Alto, CA, USA) was utilized, with the same chromatographic conditions as in the GC-MS.

### 3.5. Sensory Assessment and Panel Training

A panel of ten judges, aged 20–40, was formed for this study. These judges underwent intensive training over six months, dedicating two hours per week. During training, they received instructions on describing the aromas using a coffee kit (Le Nez du Café^®^), which includes the 32 most essential sensory descriptors found in coffee. The judges were trained to identify sensory descriptors such as wood, roasts, sweets, nuts, citrus, florals, dairy, and other descriptors of coffee aroma. Subsequently, they were trained with CB (CB) samples that were placed in dark containers labeled with a three-digit code to prevent bias [23,24].

At the end of the training period, the panelists were subjected to a classification evaluation of recognizing and classifying the different samples according to their smell. Five panelists who received additional training in olfactometry with a test solution prepared in the laboratory and who subsequently received CB samples were analyzed from this test. Five trained judges analyzed the CB samples. Each judge conducted a complete analysis at 18 min intervals, recording retention times (RTs), aroma descriptors, and intensity (0–3; 0 not detected–3 highest intensity) of potential compounds with sensory activity. The judges recorded each perceived cue’s scent descriptors, retention times, and scent intensities. The modified frequency (MF) in Equation (2) was calculated using the provided data, where F (%) represents the detection frequency of an aromatic attribute expressed as a percentage, and I (%) is the average intensity, also expressed as a percentage. These parameters make it possible to estimate the most essential odorant compounds that have the most significant impact on the aroma of CB [19,25,26,27,28].

(2) $$\%MF=\sqrt{\%I*\%F}$$

Obtaining informed consent was achieved by presenting the panelists with a statement, which read: “I acknowledge that my responses will be kept confidential, and I agree to participate in this evaluation of coffee samples”. A written affirmative response was a prerequisite for participation, and the panelists had the option to withdraw from the evaluation at any time without offering any reasons.

### 3.6. Sample Preparation Methodology

#### 3.6.1. HS-SPME Technique Optimization

A Box–Behnken experimental design was conducted using Minitab 18.1 software, generating 45 experiments. Four operating variables were used in this design: extraction time (10–50 min), sample volume (2–6 mL), conditioning time (5–15 min), and fiber type (DVB/PDMS/CAR, DVB/PDMS, or PDMS) (Table S1). The temperature remained constant at 37 °C as we sought to analyze compounds with sensory activity considering the mouth’s temperature. This temperature value has been used in various studies on aroma in the food industry [2,29]. All analyses were performed using GC-O-FID by applying the chromatographic conditions described in Section 3.4.

#### 3.6.2. Verification of Aroma Extraction Methodology Using HS-SPE

For the extraction of volatile compounds from the CB, the HS-SPE technique was used following the optimal conditions previously established to extract volatile compounds from wine samples [12]. Since the HS-SPE technique has proven its effectiveness in obtaining representative extracts from complex matrices such as wine, a study of the critical variables in the process was carried out [11,28].

This verification was performed because of the differences between CB and wine. The uniqueness of the CB matrix may require adjustment and validation of extraction parameters, specifically for this beverage. This approach guarantees the SPE technique’s accuracy and applicability by recognizing the samples’ particularities and ensuring the representativeness of the extracted volatile compounds.

Evaluations were carried out to investigate the effect of aroma extraction time, considering four different durations: 1, 2.5, 4, and 6 h. In addition, variables such as purge gas flow, with 80, 100, and 120 mL/min options, and dichloromethane/methanol solvent ratio, with 95/5 and 90/10 alternatives, were examined. The primary purpose of this study is to determine the optimal conditions for generating favorable results. The subsequent analysis used the same chromatographic conditions to optimize the HS-SPME technique.

The application of HS-SPE to obtain a representative sample of CB aroma arises from the need to complement the HS-SPME method. Because HS-SPME operates at equilibrium, specific volatile compounds, particularly polar compounds, are not accurately perceived, as observed in previous investigations. These studies demonstrated that the extraction of volatiles through nitrogen purging over the surface of a beverage, followed by adsorption on an SPE cartridge with 400 mg of LiChrolut, is effective. This cartridge was chosen based on the existing literature that has validated its use and effectiveness for this purpose [11,13,25]. The headspace variant of SPE was utilized via a configuration analogous to that employed in prior investigations, followed by elution of the compounds in the SPE cartridge with a ratio of dichloromethane to methanol to obtain a representative aroma extract in the solvent for GC-O analysis [30]. Therefore, the employment of this variant of SPE offers a more comprehensive characterization of CB aroma.

### 3.7. Identification of Compounds with HS-GC-O-MS Sensory Activity

Tentative identification of the sensory-active compounds was carried out using a comparative analysis of the mass spectra, aromatic profiles, and retention indices of Kovats obtained experimentally and those previously reported in the literature, databases, and libraries such as NIST.

## 4. Conclusions

Research on aromatic compounds in CB coffee using GC-O-MS through solid-phase extraction (HS-SPE) and headspace solid-phase microextraction (HS-SPME) has yielded significant and complementary results. HS-SPME identified a broader range of volatile compounds, whereas HS-SPE provided information that HS-SPME could not capture. This duality offers a comprehensive understanding of CB aromas and maximizes the information obtained by considering them complementary rather than competitive techniques. These methodologies thoroughly analyze the aromatic compounds crucial for sensory perception. The HS-SPE system is effective and adaptable to the complex matrix of CB. The optimization of HS-SPME has led to an efficient and sensitive methodology that allows for a complete profile of volatile compounds.

This approach enabled the identification of compounds with a high contribution to aroma. For HS-SPME, compounds such as 1,5-octadien-3-one, 5-methylfuran-2-carbaldehyde, 2-ethyl-3,5-dimethylpyrazine, and benzaldehyde were identified with descriptors including floral, caramel, sweet, and almond. In contrast, HS-SPE identified compounds such as 2,6-dimethylpyrazine, 2-ethyl-6-methylpyrazine, furaneol, and furfuryl alcohol, with descriptors such as chocolate, floral, coffee, and caramel. Consequently, the CB samples exhibited an aroma profile dominated by floral and sweet notes, consistent with the literature [1,5].

The overall conclusion highlights the significant contribution of both HS-SPE and HS-SPME techniques in understanding the aromatic profiles of CB coffee. Additionally, the applicability, practicality, and short analysis time of HS-SPME align with the principles of green chemistry, thereby emphasizing its efficiency and sustainability. This opens the possibility for future comparative studies and additional methodologies for aroma reconstruction through the quantification of the identified compounds, providing a solid foundation for advancing knowledge in CB aroma.

## Figures and Tables

**Table 1:** Results of extraction time verification (total area and number of peaks obtained when evaluating the extraction times).

Extraction Time (h)	Total Area	Number of Peaks
1	392.0	38
2.5	435.7	42
4	735.1	62
6	907.0	43

**Table 2:** Results of HS-SPE extraction flow verification (total area and number of spikes obtained by evaluating the extraction times).

Flow (mL/min)	Total Area	Number of Peaks
80	745.9	60
100	783.0	60
120	789.1	61

**Table 3:** Results of the verification of the HS-SPE extraction solvent ratio (total area and number of peaks obtained when evaluating extraction times).

Solvent Ratio	Total Area	Number of Peaks
95/5	735.1	62
90/10	418.4	37

**Table 4:** Compounds extracted using HS-SPME and HS-SPE tentatively identified via GC-O-MS with their calculated retention index (RI(E)), library retention index (RI), CAS, percentage of modified frequencies (%MF), and aroma descriptor [9,17]. N.I: The compound shows sensory activity, but it was not possible to make the proper identification; N.D: sensory activity was felt with a single sample preparation technique; much of the data were obtained in: http://www.thegoodscentscompany.com/index.html, accessed on 11 March 2023.

Compound	CAS	HS-SPME		HS-SPE		RI	Aroma Descriptor
		MF	RI(E)	MF	RI(E)		
N.I	N.I	77.5	737.8	N.I	N.I	N.I	Green, sweet, vanilla, smooth
butanal	123-72-8	58.9	886.4	N.D	N.D	886	Sweet
1,5-Octadien-3-one	65213-86-7	73.0	996.9	N.D	N.D	981	Floral
phenylacetaldehyde	122-78-1	63.2	1040.8	N.D	N.D	1048	Floral, caramel, green
pyrazine	290-37-9	49.0	1201.0	N.D	N.D	1219	Walnut, spicy, sweet corn, toasted
2-(methoxymethyl)furan	13679-46-4	28.3	1231.9	N.D	N.D	1251	Coffee
2-methyloxolan-3-one	3188-00-9	73.0	1268.7	N.D	N.D	1269	Sweet
2-Methylpyrazine	109-08-0	44.7	1269.5	57.7	1285.4	1267	Chocolate
N.I	N.I	60.6	1305.1	N.I	N.I	N.I	Floral, sweet, woody, burnt
2,5-dimethylpyrazine	109-08-0	54.2	1319.9	51.6	1308.6	1320	Green, wood, tan
2,6-dimethylpyrazine	108-50-9	N.D	N.D	60.6	1321.0	1322	Chocolate
2-ethyl-3,5-dimethylpyrazine	13925-07-0	70.5	1329.3	N.D	ND	1328	Toasted, sweet
2-ethylpyrazine	13925-00-3	40.0	1341.7	40.7	1338.0	1334	Toasted, roasted
2-ethyl-6-methylpyrazine	13925-03-6	N.D	N.D	51.6	1368.0	1363	Floral, green
2-ethyl-5-methylpyrazine	13360-64-0	63.2	1382.9	75.3	1396.7	1387	Coffee, green
linalool oxide/(*Z*)-linalool oxide	1365-19-1/5989-33-3	83.7	1422.2	85.6	1451.2	1451	Floral, sweet
3-ethyl-2,5-dimethylpyrazine	13360-65-1	20.0	1442.3	N.D	N.D	1447	Toasted
acetic acid	64-19-7	87.6	1457.5	85.0	1466.4	1460	Sharp, pungent, sour, vinegar
furfural	98-01-1	52.9	1463.8	50.0	1474.3	1473	Sweet, woody, bready, caramel
2,3-diethyl-5-methylpyrazine	18138-04-0	52.9	1481.8	50.0	1494.3	1492	Green, tan, wood
ethanone, 1-(2-furanyl)-	1192-62-7	69.3	1503.3	N.D	N.D	1499	Sweet, cocoa
pyrrole	109-97-7	N.D	N.D	40.0	1507.2	1507	Dust, humidity
benzaldehyde	100-52-7	75.3	1524.4	N.D	N.D	1528	Sweet, bitter, almond
furan-2-ylmethyl acetate	623-17-6	52.9	1537.5	43.0	1533.2	1531	Sweet, savory, banana
linalool	78-70-6	100.0	1550.9	100.0	1550.9	1547	Woody, green, citric, floral, green, patchouli
5-methylfuran-2-carbaldehyde	620-02-0	75.3	1567.5	N.D	N.D	1570	Sweet, caramel, pungent
furan-2-ylmethyl propanoate	623-19-8	81.6	1594.2	51.6	1599.7	1602	Sweet savory, green, banana, coffee
2-(furan-2-methyl)furan	1197-40-6	68.3	1600.4	65.4	1609.7	1615	Toasted
butyrolactone	96-48-0	49.0	1629.2	68.3	1631.7	1632	Caramelized
furfuryl Alcohol	98-00-0	N.D	N.D	44.7	1662.1	1666	Earthy, sulfurous
2-thiophenecarboxaldehyde	98-03-3	49.0	1717.2	48.9	1685.7	1684	Walnut, hazelnut, walnut
ethanone, 1-(2-thienyl)	88-15-3	77.5	1747.5	54.8	1755.5	1763	Sweet, earthy, green, floral
2-buten-1-one, 1-(2,6,6-trimethyl-1,3-cyclohexadien-1-yl)-	23696-85-7	63.2	1793.9	N.D	N.D	1801	Tasty, coffee
1H-Pyrrole, 1-(2-furanylmethyl)-	1438-94-4	79.6	1832.6	75.9	1811.9	1824	Phenolic, smoky, spicy
phenol, 2-methoxy-	90-05-1	60.6	1862.8	60.6	1856.2	1861	Floral, rose, phenolic.
benzyl alcohol	100-51-6	58.9	1881.2	N.D	N.D	1870	Sweet, caramel
2-cyclopenten-1-one, 3-ethyl-2-hydroxy	21835-01-8	61.1	1893.3	N.D	N.D	1894	Herbal, hospital
2-Thiophenemethanol	636-72-6	31.6	1908.1	N.D	N.D	1930	Coffee, roasted
furan, 2,2′-[oxybis(methylene)]bis-	4437-22-3	51.6	1971.7	71.8	1949.8	1986	Fusty, coffee
1H-pyrrole-2-carboxaldehyde	1003-29-8	77.5	2035.3	N.D	N.D	2030	Earthy, sulfurous
furaneol	3658-77-3	58.9	N.D	56.6	2038.0	2039	Caramel, sweet
4-ethylguaiacol	2785-89-9	73.0	N.D	44.7	2070.2	2055	Vanilla, sweet
2-methoxy-4-vinylphenol	7786-61-0	63.2	2096.6	40.8	2163.7	2188	Phenolic, spicy, smoky, woody, powdery
indole	120-72-9	N.D	N.D	36.5	2405.1	2448	Floral
vanillin	121-33-5	28.3	2526.7	31.6	2467.0	2566	Vanilla, sweet

**Table 5:** Compounds exclusively identified in each sample preparation technique via GC-O-MS with their percentage of modified frequencies (%MF) and aroma descriptors [14].

**HS-SPE**		
**Compound**	**%MF**	**Aroma Descriptor**
2,6-dimethylpyrazine	60.6	Chocolate
2-ethyl-6-methylpyrazine	51.6	Floral, green
Pyrrole	40.0	Sweet
furfuryl Alcohol	44.7	Coffee
Furaneol	56.6	Caramel, sweet
4-ethylguaiacol	44.7	Vanilla, sweet
Indole	36.5	Floral
**HS-SPME**		
**Compound**	**%FM**	**Aroma Descriptor**
Butanal	58.9	Sweet
1,5-octadien-3-one	73.0	Floral
Phenylacetaldehyde	63.2	Floral, caramel, green
Pyrazine	49.0	Walnut, spicy, sweet corn, toasted
2-(methoxymethyl)furan	28.3	Coffee
2-methyloxolan-3-one	73.0	Sweet
2-ethyl-3,5-dimethylpyrazine	77.5	Toasted, sweet
3-ethyl-2,5-dimethylpyrazine	20.0	Toasted
ethanone, 1-(2-furanyl)-	69.3	Sweet, cocoa
Benzaldehyde	75.3	Sweet, almond
5-methylfuran-2-carbaldehyde	75.3	Sweet, caramel, pungent
2-buten-1-one, 1-(2,6,6-trimethyl-1,3-cyclohexadien-1-yl)-	63.2	Sweet, earthy, green, floral
benzyl alcohol	58.9	Floral, rose, phenolic
2-Thiophenemethanol	31.6	Coffee, roasted
2-cyclopenten-1-one, 3-ethyl-2-hydroxy	61.1	Sweet, caramelized, maple
1H-pyrrole-2-carboxaldehyde	25.8	Fusty, coffee

## Data Availability

The data are contained in the article and the Supplementary Material.

## Supplementary Materials

The following supporting information can be downloaded at: https://www.mdpi.com/article/10.3390/molecules29163791/s1. Table S1: Operational variables to optimize solid-phase microextraction in its headspace mode (HS-SPME) with their respective injection order obtained from the Box-Behnken design of experiments. Table S2: Results of the response parameters (total area and number of peaks) from solid- phase microextraction in its headspace mode (HS-SPME) (Agilent), with manual injection into the Agilent 6890N gas chromatograph with a flame ionization detector (FID), varying the extraction time, equilibrium time, fiber type, and sample volume based on the experimental design shown in Table 5, with two types of data handling: analysis of the entire chromatogram and segmented analysis (Segment 1: [0–15] min; Segment 2: (15–28] min; and Segment 3: (28–52] min). Table S3: Retention times (RT) and Kovats retention indices (RI) of the analyzed alkanes (C7–C30). Table S4: Compounds with Aroma that Could Not Be Identified (KI: Experimental Kovats Retention Index, MF: Modified Frequency, Compound: Tentative Compound Identified, KI(i): Theoretical Kovats Retention Index for Compound i, i: Compound i identified by NIST19.
