# Supplementary material for: A Comparative Analysis of Cold Brew Coffee Aroma Using the Gas Chromatography–Olfactometry–Mass Spectrometry Technique: Headspace–Solid-Phase Extraction and Headspace Solid-Phase Microextraction Methods for the Extraction of Sensory-Active Compounds
Source: Molecules. 2024 Aug 10;29(16):3791. doi: 10.3390/molecules29163791 (PMC11357319; doi:10.3390/molecules29163791)
Supplement: Supplementary file 1 [file molecules-29-03791-s001.zip › molecules-3062499-supplementary.pdf]

Supplementary Materials

Table S1: Operational variables to optimize solid-phase microextraction in its headspace mode (HS-SPME) with their respective injection order obtained from the Box-Behnken design of experiments. 2

Table S2: Results of the response parameters (total area and number of peaks) from solid-phase microextraction in its headspace mode (HS-SPME) (Agilent), with manual injection into the Agilent 6890N gas chromatograph with a flame ionization detector (FID), varying the extraction time, equilibrium time, fiber type, and sample volume based on the experimental design shown in Table 5, with two types of data handling: analysis of the entire chromatogram and segmented analysis (Segment 1: [0-15] minutes; Segment 2: (15-28] minutes; and Segment 3: (28-52] minutes). 3

Table S3: Retention times (RT) and Kovats retention indices (RI) of the analyzed alkanes (C7-C30) 4

Table S4: Compounds with Aroma that Could Not Be Identified (KI: Experimental Kovats Retention Index, MF: Modified Frequency, Compound: Tentative Compound Identified, KI(i): Theoretical Kovats Retention Index for Compound i, i: Compound i identified by NIST19 5

Table S1: Operational variables to optimize solid-phase microextraction in its headspace mode (HS-SPME) with their respective injection order obtained from the Box-Behnken design of experiments.

Order of Experiments	Operational Variables			
	Extraction Time (min)	Sample Volume (mL)	Conditioning Time (min)	Fiber Type
**1**	10	4	5	DVB/PDMS/CAR
**2**	30	6	15	PDMS
**3**	50	4	15	DVB/PDMS/CAR
**4**	30	2	5	DVB/PDMS
**5**	50	2	10	PDMS
**6**	30	4	10	PDMS
**7**	10	4	5	PDMS
**8**	30	6	5	PDMS
**9**	30	2	5	PDMS
**10**	10	4	5	DVB/PDMS
**11**	30	2	15	PDMS
**12**	30	6	15	DVB/PDMS/CAR
**13**	30	2	5	DVB/PDMS/CAR
**14**	50	4	15	PDMS
**15**	50	6	10	DVB/PDMS/CAR
**16**	30	6	5	DVB/PDMS
**17**	10	6	10	PDMS
**18**	50	4	5	DVB/PDMS
**19**	30	2	15	DVB/PDMS/CAR
**20**	10	6	10	DVB/PDMS
**21**	30	6	5	DVB/PDMS/CAR
**22**	30	4	10	DVB/PDMS
**23**	50	2	10	DVB/PDMS
**24**	30	4	10	DVB/PDMS/CAR
**25**	30	4	10	PDMS
**26**	10	2	10	PDMS
**27**	10	4	15	DVB/PDMS
**28**	10	2	10	DVB/PDMS/CAR
**29**	50	4	15	DVB/PDMS
**30**	30	4	10	DVB/PDMS
**31**	30	4	10	PDMS
**32**	30	4	10	DVB/PDMS/CAR
**33**	50	4	5	PDMS
**34**	30	4	10	DVB/PDMS/CAR

**35**	50	6	10	DVB/PDMS
**36**	10	4	15	PDMS
**37**	30	4	10	DVB/PDMS
**38**	10	2	10	DVB/PDMS
**39**	30	6	15	DVB/PDMS
**40**	10	6	10	DVB/PDMS/CAR
**41**	10	4	15	DVB/PDMS/CAR
**42**	50	4	5	DVB/PDMS/CAR
**43**	50	6	10	PDMS
**44**	30	2	15	DVB/PDMS
**45**	50	2	10	DVB/PDMS/CAR

Table S2: Results of the response parameters (total area and number of peaks) from solid-phase microextraction in its headspace mode (HS-SPME) (Agilent), with manual injection into the Agilent 6890N gas chromatograph with a flame ionization detector (FID), varying the extraction time, equilibrium time, fiber type, and sample volume based on the experimental design shown in Table 5, with two types of data handling: analysis of the entire chromatogram and segmented analysis (Segment 1: [0-15] minutes; Segment 2: (15-28] minutes; and Segment 3: (28-52] minutes).

**Order of Experiments**	**Response Parameters**							
	**Complete Chromatograph**		**Segment 1**		**Segment 2**		**Segment 3**	
	**Total Area**	**Number of Peaks**	**Total Area**	**Number of Peaks**	**Total Area**	**Number of Peaks**	**Total Area**	**Number of Peaks**
**1**	3186.3	46	1907.7	27	1251.5	19	490	10
**2**	1443.9	30	74.4	4	119.84	5	257.5	8
**3**	10621.3	112	2313	31	5572.5	44	2735.8	37
**4**	5275.3	98	1192.8	25	2329	34	1753.5	30
**5**	1583.3	37	26.4	4	83.8	5	379.5	7
**6**	1479.2	29	15.8	4	87.3	5	269.6	6
**7**	1221.5	19	24.2	3	72.1	6	143.6	4
**8**	1020.1	22	17.2	2	74	4	316.1	7
**9**	2020.7	27	24.5	3	78.6	6	485.5	8
**10**	2336.6	41	1275.1	27	1025.3	17	488	10
**11**	1419.9	28	24.8	4	68.4	3	309.6	6
**12**	8183.3	108	2174.6	30	4404.8	45	1603.9	35
**13**	5385.7	64	1978.8	26	3242.7	39	416	10
**14**	847.7	15	25.4	2	74.3	5	272.975	8
**15**	10816.8	118	2383.8	32	6019.4	45	2413.6	41
**16**	4594.1	71	1427.2	26	2750.2	34	396.5	9
**17**	623.7	27	50.3	2	92.4	4	238.6	8
**18**	6969.7	110	1455.3	32	3543.9	35	1970.5	35

**19**	9007.4	112	2320.7	30	4738.4	42	1948.3	40
**20**	4800.6	82	1272.6	25	1943.3	30	1584.7	28
**21**	9273	115	2380.6	33	4685.8	45	2206.6	40
**22**	5642.9	100	1229.6	25	2409.8	34	2003.5	39
**23**	7086.9	111	1341.1	25	3427.2	40	2318.6	40
**24**	8420.8	113	2347.7	32	4194.3	43	1878.8	40
**25**	1519.9	39	46.9	3	95.1	6	403.9	8
**26**	121	7	25.1	1	60.1	3	0	0
**27**	4413.7	70	1306.8	25	1657.5	30	1449.4	28
**28**	3296.4	49	1694.3	29	1569.1	24	500	10
**29**	7154.3	105	1374.2	25	3435.6	40	2344.5	40
**30**	5984	102	1368.9	25	2821.3	40	1793.8	28
**31**	538.4	16	27.5	1	77.2	5	400	8
**32**	5943.8	72	2151.4	31	3558.4	34	520	10
**33**	966.1	37	40.2	3	116.9	5	313.3	8
**34**	6131.2	75	2025.9	30	3801.8	36	535	14
**35**	7194.3	104	1449.4	26	3617.4	40	2127.5	37
**36**	89.8	6	14.5	1	24.7	2	21.8	1
**37**	6117.5	108	1390.5	25	3030.3	42	1672.2	27
**38**	4587.6	74	1335.4	25	1817.1	34	1435.1	25
**39**	6121.9	104	1372.7	25	2987.7	39	1761.5	27
**40**	3980.8	80	1269.4	30	1881.2	39	830.2	25
**41**	5288	82	2079.2	30	2186.6	39	1022.2	28
**42**	9936.9	116	2198.8	30	5540.7	45	2197.4	43
**43**	920.6	35	51.1	3	108.4	6	382.6	8
**44**	6232.4	106	1304	25	3192.2	40	1736.2	40
**45**	9747.6	115	2037.4	30	5726.3	44	1983.9	28

Table S3: Retention times (RT) and Kovats retention indices (RI) of the analyzed alkanes (C7-C30)

#C	Alcano	RT (min)	RI (min)
**7**	Heptane	1.275	700
**8**	Octane	2.285	800
**9**	Nonane	2.561	900
**10**	Decane	3.558	1000
**11**	Undecane	5.025	1100
**12**	Dodecane	5.72	1200
**13**	Tridecane	8.78	1300
**14**	Tetradecane	12.303	1400
**15**	Pentadecane	15.925	1500
**16**	Hexadecane	19.567	1600

**17**	Heptadecane	23.028	1700
**18**	Octadecane	26.388	1800
**19**	Nonadecane	32.716	1900
**20**	Eicosane	35.697	2000
**21**	Heneicosane	38.274	2100
**22**	Docosane	40.198	2200
**23**	Tricosane	40.647	2300
**24**	Tetracosane	41.776	2400
**25**	Pentacosane	43.147	2500
**26**	Hexacosane	44.393	2600
**27**	Heptacosane	45.541	2700
**28**	Octacosane	46.624	2800
**29**	Nonacosane	47.651	2900
**30**	Triacontane	48.637	3000

Table S4: Compounds with Aroma that Could Not Be Identified (KI: Experimental Kovats Retention Index, MF: Modified Frequency, Compound: Tentative Compound Identified, KI(i): Theoretical Kovats Retention Index for Compound i, i: Compound i identified by NIST19

**KI**	**MF**	**Compound**					
		**1**	**KI(1)**	**2**	**KI(2)**	**3**	**KI(3)**
737.8	77.5	The Kovats retention index did not find a signal in the corresponding mass spectra.					
1305.1	60.6	7-methyl-Z-tetradecen-1-ol acetate	2108	cyclopropanepentanoic acid, 2-undecyl-, methyl ester, trans-	2444	Cyclopropanepentanoic acid, 2-undecyl-, methyl ester, trans-	2445
